# A Self-Limiting Electro-Ablation Technique for the Top-Down Synthesis of Large-Area Monolayer Flakes of 2D Materials

**DOI:** 10.1038/srep28195

**Published:** 2016-06-21

**Authors:** Saptarshi Das, Mrinal K. Bera, Sheng Tong, Badri Narayanan, Ganesh Kamath, Anil Mane, Arvydas P. Paulikas, Mark R. Antonio, Subramanian K. R. S. Sankaranarayanan, Andreas K. Roelofs

**Affiliations:** 1 Department of Engineering Science and Mechanics & Material Research Institute, Pennsylvania State University, State College, 16803, USA; 2 DUBBLE-CRG, European Synchrotron Radiation Facility, CS40220, 38043 Grenoble Cedex 9, France; 3 Nanoscience and Technology Division, Argonne National Laboratory, Argonne, Illinois, 60439, USA; 4 Department of Chemistry, University of Missouri, Columbia, Missouri, 65211, USA; 5 Energy Science Division, Argonne National Laboratory, Argonne, Illinois, 60439, USA; 6 Material Science Division, Argonne National Laboratory, Argonne, Illinois, 60439, USA; 7 Chemical Sciences and Engineering Division, Argonne National Laboratory, Argonne, Illinois, USA

## Abstract

We report the discovery of an electrochemical process that converts two dimensional layered materials of arbitrary thicknesses into monolayers. The lateral dimensions of the monolayers obtained by the process within a few seconds time at room temperature were as large as 0.5 mm. The temporal and spatial dynamics of this physical phenomenon, studied on MoS_2_ flakes using *ex-situ* AFM imaging, Raman mapping, and photoluminescence measurements trace the origin of monolayer formation to a substrate-assisted self-limiting electrochemical ablation process. Electronic structure and atomistic calculations point to the interplay between three essential factors in the process: (1) strong covalent interaction of monolayer MoS_2_ with the substrate; (2) electric-field induced differences in Gibbs free energy of exfoliation; (3) dispersion of MoS_2_ in aqueous solution of hydrogen peroxide. This process was successful in obtaining monolayers of other 2D transition metal dichalcogenides, like WS_2_ and MoTe_2_ as well.

For the last several decades, silicon has been the DNA of our technological evolution. And, now with the emergence of the era of the Internet of Things (IoT)^1^, novel materials need to be mutated into the genetics of modern technologies in order to meet the ever increasing demands of new functionalities. In this context, interest in two-dimensional (2D) transition metal dichalcogenides (TMDs) is rapidly spreading across all scientific and engineering disciplines as a result of their physical properties, like room temperature quantum Hall effect^2^, charge-density waves^3^, high-temperature superconductivity^4^, superfluidity^5^, and high carrier mobility^6^ in the monolayer limit. In fact, field-effect transistors, gas sensors, bio-detectors, mechanical resonators, optical modulators and energy-harvesting devices with superior performances have already been demonstrated based on monolayers of different TMDs^7^,^8^,^9^,^10^,^11^,^12^,^13^. The early successes of 2D monolayers have attracted the investment of millions of dollars in research and development by several government and private sector organizations across the globe^14^,^15^. It is, therefore, important to develop energy-efficient techniques to obtain 2D monolayers.

Most of the contemporary research in the field has focused on 2D monolayers obtained through micromechanical exfoliation of naturally occurring single crystals. In spite of being scalable, fast, and cost effective, this technique has poor yield and also lacks reproducibility. Chemical vapor deposition (CVD) is the most widely used bottom-up technique to grow 2D-monolayers over large areas in a sustainable and reproducible way. For example, large-area monolayer MoS_2_ has been grown by thermal decomposition of thiomolybdates^16^ and sulfurization of metallic Mo or molybdenum chloride or molybdenum oxide^17^,^18^,^19^,^20^. However, CVD processes require high temperatures (600–1000 °C) and long processing times (several hours). Other bottom-up techniques like molecular beam epitaxy (MBE), pulsed laser deposition and atomic layer deposition (ALD) are still under development for synthesizing monolayers of all the known 2D materials. Several top-down approaches have also been adopted based on bulk liquid phase chemical and electrochemical exfoliation of MoS_2_. It is well known that selected alkali metal ions (e.g., Li, Na, and K) can intercalate inside the interlayer space in multilayer MoS_2_ resulting in an expanded lattice, which can then be used to exfoliate single sheets of MoS_2_ by ultrasound-assisted hydration processes^21^,^22^. However, the long intercalation time (several days), low monolayer yield (less than 10%), and disintegration of monolayer flakes into sub-micron size particles are the major limitations of this technique. Recently, oxidant-promoted exfoliation of MoS_2_ was achieved with hydrogen peroxide (H_2_O_2_)^17^,^18^. The spontaneous exfoliation using mixed solvents containing H_2_O_2_ took 10 hrs and resulted in 2–5 μm MoS_2_ monolayer flakes^23^. In contrast, electrochemical exfoliation using H_2_O_2_ required 10 V for 2 hrs and resulted in only a 7% yield of monolayers with lateral sizes in the range of 5–50 μm^24^.

With the prospect of advancing the field in mind, we demonstrate an electrochemical technique, hereafter referred to as the electro-ablation (EA) technique, for room-temperature synthesis of monolayers of semiconducting TMDs on a conductive substrate. A comparison of the EA technique with other state-of-the-art techniques related to the synthesis of MoS_2_ monolayers (See Table S1 in the Supporting Information (SI)) reveals three clear advantages: (1) fast synthesis (5–60 seconds); (2) energy efficiency; and (3) high yields.

Figure 1a describes, schematically, the two-step synthesis of MoS_2_ monolayers using the EA technique. In the first step, multilayer MoS_2_ flakes are adhered on top of a conductive TiN substrate using micromechanical exfoliation. In the second, a conventional electrochemical setup (middle panel of Fig. 1a) is used to apply a positive electrode potential to the substrate dipped into an aqueous electrolyte solution (1 M LiCl) for a short period of time (5–60 seconds). On inspection of the substrate, we found that all of the top layers from the individual multilayer MoS_2_ flakes were removed. The substrate was left with ultrathin uniform layers of MoS_2_. Both the choice of the conductive substrate and the aqueous electrolyte solution are crucial for the success of the EA technique. These issues are discussed in a subsequent section.

Figure 1b shows the optical micrographs of exfoliated MoS_2_ flakes before and after the EA treatment. The optical contrast suggests that the untreated portion of the substrate is covered with multilayered MoS_2_ flakes of variable thicknesses, whereas the treated portion is covered with flakes of uniform thickness. Raman shift and photoluminescence characterization (vide infra) confirm that these uniform layers are, in fact, monolayers of MoS_2_. The lateral dimension of the monolayer flakes obtained through the EA technique has no fundamental limitation since it is dependent on the size of the multilayer flakes obtained from micromechanical exfoliation. In this context, a thermally-activated, solvent-mediated, and ultra-sound assisted micromechanical exfoliation process (Figure S2 in the SI) was developed in order to increase the density of large-area flakes and, hence, the coverage of the substrate (75–80%).

Figure 1c shows the AFM images and the height histograms of the MoS_2_ flakes before and after the EA treatment. Multiple peaks positioned at approx. 12, 16, 19 and 34 nm are found in the height histogram (middle panel of Fig. 1c) corresponding to the AFM image of the as-exfoliated MoS_2_ flakes (the substrate peak is centered at zero). This random height distribution is a natural outcome of the mechanical exfoliation technique. However, after the EA treatment, all of these peaks collapsed into a single peak at 2.5 nm. This indicates that the initial flake thickness is inconsequential for the EA technique, which transforms any thicknesses of multilayered MoS_2_ flakes into layers of 2.5 nm thickness. In fact, we were able to planarize MoS_2_ flakes with thicknesses in the range of hundreds of nm down to 2.5 nm using the EA technique. The 2.5 nm thickness of the EA-treated MoS_2_ monolayer is due to the time-dependent weak etching of the exposed part of the conducting TiN substrate (vide infra) that increases the effective height of the monolayer. The FWHM (full width at half max) of approx. 1 nm associated with the monolayer peak at 2.5 nm (and also with the multilayer peaks) in the AFM height histogram is directly attributable to the TiN substrate (with an essentially identical FWHM) rather than non-uniformity of the flakes.

In order to ascertain the number of MoS_2_ layers left on the EA-treated substrate that corresponds to the thickness of 2.5 nm, we performed Raman spectroscopy and photoluminescence (PL) measurements. Figure 2a shows the Raman shift data of a MoS_2_ flake before and after the EA treatment. The separation between the A_1g_ and E^1^_2g_ peaks is reduced from 26.7 cm^−1^ to 20.6 cm^−1^. This suggests that the multilayer MoS_2_ flake has been converted to monolayer MoS_2_^25^. Moreover, the PL data, obtained from EA-treated MoS_2_ flakes, shown in Fig. 2b, exhibit a peak corresponding to a bandgap energy *E*_G_ = 1.86 eV. This is a clear fingerprint of monolayer MoS_2_^26^. The uniformity of the MoS_2_ layers obtained from the EA treatment was studied through the Raman mapping of a representative flake as shown in Fig. 2c. The distribution of the peak separation has a maximum at approx. 20.6 cm^−1^ corresponding to monolayer MoS_2_. Because the width of this distribution (approx. 1.0 cm^−1^) is less than the resolution of the Raman instrument (1.6 cm^−1^), we can confirm that the monolayer MoS_2_ flakes are uniform^27^. We also performed XPS measurements on the MoS_2_ flakes before and after the application of the EA technique, as shown in Section S3 of the SI. The presence of S-2p peaks and Mo-3d peaks at the same energies before and after the EA treatment shows that the technique has converted the multilayers of MoS_2_ to monolayers without affecting their composition or forming any other products of Mo and/or S.

Figure 3 shows the temporal and spatial dynamics of the EA technique for three different flake thicknesses using the *ex-situ* AFM images, the initial and final height distributions, and the Raman shifts. As shown in Fig. 3a, a 12 nm thick flake was fully converted into a monolayer in less than 2 s. For comparison, 29 nm and 56 nm thick flakes required less than 5 s and 20 s to be converted into monolayers as shown in Fig. 3b,c, respectively (note that we only have the *ex-situ* AFM data for 1, 2, 5, and 20 s). The initial and final height histograms and the corresponding Raman shifts shown in Fig. 3 suggest that the self-limiting EA technique yields monolayer MoS_2_. This is one of two distinct features of the EA technique. First, as discussed earlier, at the end of the EA treatment, the flakes are reduced to monolayers irrespective of their initial thicknesses. Second, the electrochemical processes responsible for the conversion of multilayer flakes into monolayers begin at the edges and progressively remove the inner areas with time. In fact, the thickness of the undispersed portion of the flake at any given point in time remains constant and the same as the initial flake thickness (Fig. S4 in the SI). That is, the bottommost layers of all the flakes are left unaltered by the EA treatment. This is precisely why the EA treatment provides monolayers of MoS_2_. Such a self-limiting electrochemical process is an outcome of several essential contributing factors. These include strong covalent bonding interactions of the monolayer MoS_2_ with the substrate, weak van der Waals interactions between the individual layers of MoS_2_, and electric-field induced differences in the Gibbs free energy of solvation of MoS_2_ in aqueous electrolytes (vide infra). The effective thickness of the monolayer flakes (d_eff_) increases monotonically with the processing time due to the weak etching of the TiN substrate (d_eff_ = d_ML_ + d_etch_, where d_ML_ is the true monolayer thickness of MoS_2_ and d_etch_ is the thickness of uncovered TiN substrate etched during the EA treatment). As expected d_etch_ increases with time. It is obvious that thinner flakes are ablated to monolayers faster than the thicker flakes because less volume of the material needs to be removed. Also, once a multilayer flake is converted to a monolayer one, it remains unaltered (except for the fact that its effective thickness increases with time due to the etching of the substrate) for the rest of the processing time. This behavior makes the EA technique self-limiting (Fig. S5 in the SI).

Finally, the underlying atomistic pathway and chemistry behind the self-limiting electrochemical ablation processes of the technique are explained in Fig. 4. Figure 4a shows differential pulse voltammetry (DPV) data of a conducting TiN substrate in aqueous electrolyte (1 M LiCl) solutions with different pH values (0.5 and 2.3). The peak at approx. 1.6 V (with respect to the Ag/AgCl reference electrode) for pH = 0.5 and the shift of the peak to a lower potential (approx. 1.5 V) at a higher pH value is consistent with the electrochemical reaction known to oxidize TiN to TiO_2_^28^,^29^,^30^, namely, TiN + 2H_2_O → TiO_2_ + 1/2N_2_ + 4H^+^ + 4e^−^. In addition to the passivation of the TiN surface to TiO_2_ at the electrode potential of 1.5 V, other reactive species form. Examination of standard electrode potentials for water and chloride ion reveals oxidation events that are also close to 1.5 V^31^ and produce reactive species, like O_2_, H_2_O_2_, Cl_2_ and other soluble products (e.g., HClO, HClO_2_, ClO_2_^−^, ClO_3_^−^, ClO_4_^−^). When the EA technique is applied on MoS_2_-coated TiN substrates, any one (or more) of these reactive species can be responsible for ablating the multilayers of MoS_2_ to monolayers and for etching the exposed surface of the TiN substrate^32^ prior to its oxidation to TiO_2_. Once the entire exposed surface is oxidized, the electrochemical reactions involved in the electro-ablation process stop. We confirmed that the EA technique is driven by electrochemical phenomena by carrying out the reactions at electrode potentials below and above the peak value of 1.5 V (pH = 2.3). We observed that the EA technique works only (Figure S6 in the SI) when the applied electrode potential is at and above the peak potential. Nothing happens at lower potentials. We also performed the EA technique on MoS_2_ flakes exfoliated on a TiN substrate already treated with the EA technique. The AFM measurements (Figure S7 in the SI) show no change in the thicknesses of the MoS_2_ layers due to EA treatment after the passivation of TiN to TiO_2_. Among the possible soluble reactive species (mentioned above) formed at the TiN surface as a result of electrochemistry, we ruled out Cl_2_ and other soluble products of the oxidation of Cl^−^ because the EA process occurs in a 0.3 M HNO_3_ electrolyte solution. We can also rule out O_2_ because MoS_2_ does not react with O_2_ at room temperature and atmospheric pressure. Hence, the most probable oxidation product is H_2_O_2_. Its electrochemical formation at the TiN surface by the application of an electrode potential of 1.5 V (pH = 2.3) and higher is responsible for not only the ablation process of MoS_2_ but also for etching and passivation of TiN to TiO_2_. Moreover, prior studies^23^ have shown that MoS_2_ flakes (especially defect and edge sites) in contact with H_2_O_2_ spontaneously undergo the reaction: MoS_2_ + 9H_2_O_2_ → MoO_2_^2+^ + 2SO_4_^2−^ + 2H^+^ + 8H_2_O. This reaction leads to the formation of smaller flakes, which undergo an exfoliation process in an H_2_O_2_ rich environment, mimicking an ablation process.

The self-limiting aspect of the EA technique arises due to differences in the binding energetics between the TiN/MoS_2_ and MoS_2_/MoS_2_ interfaces. To understand and quantify these differences, we employed density functional theory (DFT) calculations to determine whether it is energetically more favorable for a monolayer of MoS_2_ to bind with a TiN substrate rather than another layer of MoS_2_ (for details on the calculations, see section S8 in the SI). Our computed binding energies for various adsorption configurations are shown in Fig. 5. We find that the binding energy *E*_*b*_ is highest (most negative) when the MoS_2_ monolayer binds to a TiN slab containing Ti atoms in its outermost layer, such that the closest S atoms in MoS_2_ lie in the hollow sites. In this configuration, there is a strong covalent interaction between MoS_2_ and TiN with a binding energy value of −1.25 eV (see S8 in the SI for more details). In comparison, our DFT calculations show that the binding energy between two MoS_2_ monolayers is much lower (−0.16 eV) owing to weak van der Waals interactions. Considering that the binding of MoS_2_ to the underlying substrate is much stronger than that between the MoS_2_ layers, our DFT calculations suggest that it is energetically much more favorable to exfoliate MoS_2_ (all layers except the one strongly bound to the substrate) by overcoming the weak van der Waals interactions between the layers (Fig. 4b).

To provide a thermodynamic foundation for the electro-ablation phenomenon, we employed the Adaptive Bias Force method^33^ to determine the free energies of exfoliation and dispersion of MoS_2_ in water and hydrogen peroxide (H_2_O_2_), including a 30% H_2_O_2_ solution corresponding to the commercially-available peroxide reagent (see section S9 in the SI). In this method, we first placed a bilayer MoS_2_ sheet inside a supercell containing the desired solvent molecules (whose density corresponds to the experimental values), as shown in left inset of Fig. 4(c). Thereafter, an external biasing force is applied to one of the MoS_2_ layers to tangentially separate the two MoS_2_ layers by a distance *z*, i.e., the reaction coordinate [see right inset Fig. 4(c)]; this separation is performed in steps of 1 Å. At each of these steps, the value of the biasing force necessary to overcome the energy barriers (if present) is estimated by rigorous local sampling of the system conformations. The change in the free energy of the system as a function of the reaction coordinate, *z*, namely the potential of mean force for MoS_2_ exfoliation in the three different solvents (H_2_O, H_2_O_2_, 30% H_2_O_2_) are shown in Fig. 4c. The free energy of exfoliation is evaluated as the difference between the free energy of the bilayer in the solvent (Fig. 4c left inset) and the individual layers dispersed in the solvent (Fig. 4c right inset). Our calculated free energy of exfoliation of MoS_2_ in water and 30% H_2_O_2_ solution are positive and, hence, these solvents do not assist in the exfoliation and dispersion mechanism. However, a negative free energy of exfoliation is obtained as we approach the pure H_2_O_2_ limit, suggesting that exfoliation is thermodynamically feasible.

We also evaluated the pair distribution functions^34^ in Fig. 4d to understand the atomistic interactions that assist the exfoliation of MoS_2_. Figure 4d (top panel) shows that the interactions of Mo with the O atoms of H_2_O_2_ are much more pronounced in comparison to the Mo-O interactions of water and the dilute (30%) H_2_O_2_. Furthermore, the S atoms also interact more favorably with the O atoms in the pure H_2_O_2_ environment than in the dilute solution. Together, these interactions drive the electro-ablation of MoS_2_ in an H_2_O_2_-rich solution to completion, leaving behind the monolayers. The individual pair-wise distributions for Mo-O (peroxide and water) and S-O (peroxide and water) in 30% peroxide are also shown. The interactions of Mo and S are more pronounced with the oxygen of peroxide than the oxygen of water. The overall kinetics of the EA process is shown schematically in Fig. 4e,f. The choice of TiN as the conductive substrate for the EA technique is motivated by our observation that TiN appears to facilitate the oxidation of H_2_O to H_2_O_2_ as its surface is oxidized to TiO_2_.

Our initial results show that the EA technique is generic, and can be applied for the synthesis of monolayers of most of the semiconducting TMDs including, as shown in Fig. 6, WS_2_, WSe_2_ and MoTe_2_. The optical micrographs and Raman shift data show monolayer formation for WS_2_ and MoTe_2_^35^,^36^,^37^. Consistent with these findings, our DFT calculations showed that the binding energy of these monolayers with TiN is much stronger than the interlayer van der Waals interactions, similar to MoS_2_; e.g., the binding energy between WS_2_ and TiN was found to be −1.63 eV, whereas the interaction energy between consecutive layers was much lower (−0.16 eV). However, in the case of transition metal diselenides (e.g., WSe_2_), our EA technique removed the monolayer as well, despite exhibiting DFT binding energy trends similar to MoS_2_ [*E*_*b*_(WSe_2_ − TiN) = −1.7 eV; *E*_*b*_(WSe_2_ − WSe_2_) = 0.21 eV]. This suggests that for the case of diselenides, there are additional factors (apart from binding energies) that play a major role in governing the electro-ablation process. These key atomic-scale factors are likely to involve (a) the specific chemistry of selenium with H_2_O_2_, and (b) sluggish kinetics of diselenide monolayer binding with TiN as compared to the rate of removal under electrochemical conditions; regardless, a comprehensive investigation of such factors is beyond the scope of this article. Furthermore, detailed studies of the impact of the substrate and electrolyte solution are required to optimize the EA technique for other material systems and to provide a full understanding of the mechanism leading to the formation of monolayers of semiconducting TMDs. For instance, the nature of the passivation of TiN and its role in the EA technique are open-ended issues.

In summary, we have demonstrated a fast, scalable, energy-efficient and cost-effective technique (EA) to produce monolayers of semiconducting TMDs on a conducting substrate based on a self-limiting electrochemical ablation process. Although the necessity of a conducting substrate narrows the use of the monolayers formed by the EA technique for electronic device applications, it provides a direct entry to applications in catalysis, electrodes for supercapacitors, and photonic devices in which a conducting substrate is required. The EA technique can complement the CVD technique in the sense that EA can be used to remove the undesired top layers that nucleate during the CVD growth of large-area monolayers. Similarly it could complement ALD and MBE grown systems through planarization of multiple layers down to a monolayer.

## Methods

### Electrochemistry

**Figure 1:**
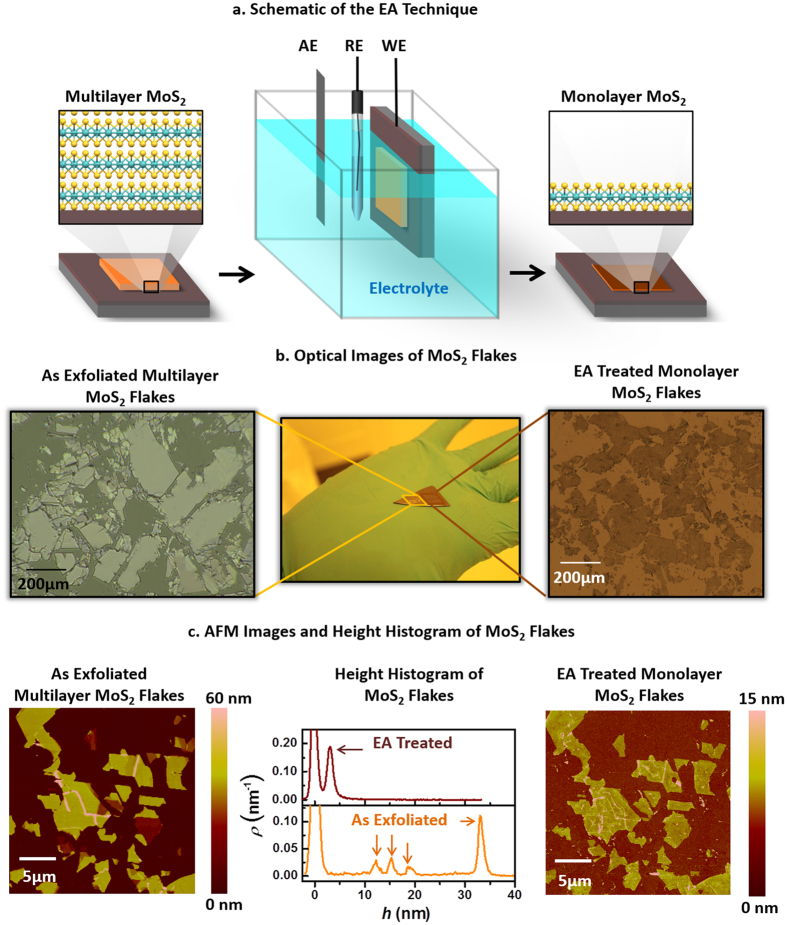
(**a**) Schematic illustrations of the electro-ablation (EA) technique which involves the exfoliation of large-area multilayered MoS_2_ flakes (left panel) on a silicon (Si) substrate coated with 100 nm conducting TiN film followed by an EA process carried out in an electrochemical cell (middle panel) resulting in the formation of monolayers of MoS_2_ (right panel). The TiN/Si substrate with exfoliated MoS_2_ flakes acts as the working electrode (WE), the Ag/AgCl half-cell acts as the reference electrode (RE), and Grafoil acts as the auxiliary electrode (AE). (**b**) Optical images of the mechanically-exfoliated large-area MoS_2_ flakes on a TiN/Si substrate before (left panel) and after (right panel) the EA treatment. (**c**) Atomic force microscopy (AFM) images of the mechanically-exfoliated MoS_2_ flakes of different thicknesses (left panel) and the uniformly-thick monolayers of MoS_2_ after the EA treatment (right panel) along with the histograms of the height profiles of both of the images (middle panel).

We performed the electrochemical ablation (EA) and differential pulse voltammetry on TiN exfoliated with multilayers of MoS_2_ and bare TiN by using an electrochemical cell shown in Fig. 1a and potentiostat from BASi Analytical Instruments, USA. We used a Ag/AgCl electrode as the reference electrode and Grafoil (from GrafTech International) of thickness 0.5 mm as the auxillary electrode. The electrode potentials reported in this article are all measured with respect to the Ag/AgCl reference electrode.

## Additional Information

**How to cite this article**: Das, S. *et al*. A Self-Limiting Electro-Ablation Technique for the Top-Down Synthesis of Large-Area Monolayer Flakes of 2D Materials. *Sci. Rep.* **6**, 28195; doi: 10.1038/srep28195 (2016).

## Supplementary Material

Supplementary Information

## Figures and Tables

**Figure 2:**
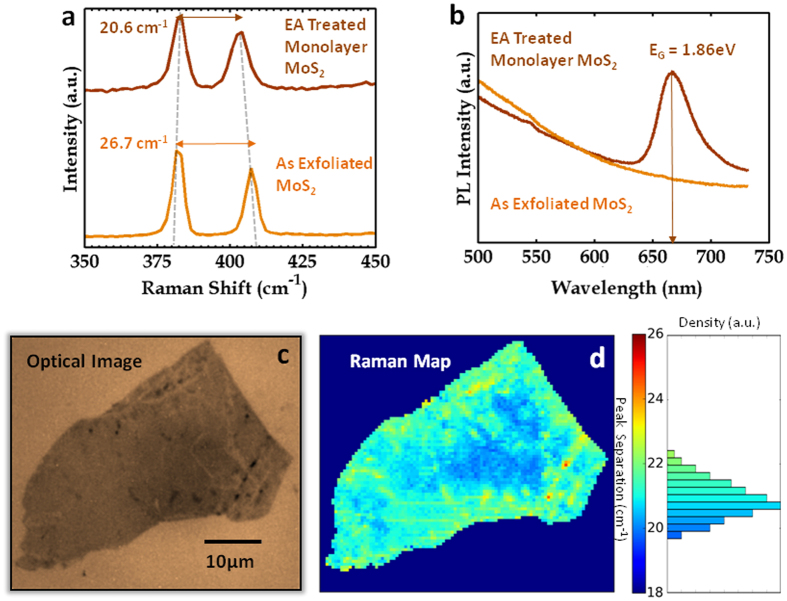
Characterization of the MoS_2_ monolayer flakes obtained through the EA technique. (**a**) Raman data and (**b**) photoluminescence data collected from the large-area mechanically exfoliated multilayers of MoS_2_ and the monolayers obtained by applying the EA technique. (**c**) Optical image and (**d**) the mapping of the Raman peak separations along with the histogram collected from a large-area monolayer of MoS_2_ obtained by the EA technique. The Raman mapping was done at the resolution of 0.5 μm × 0.5 μm.

**Figure 3:**
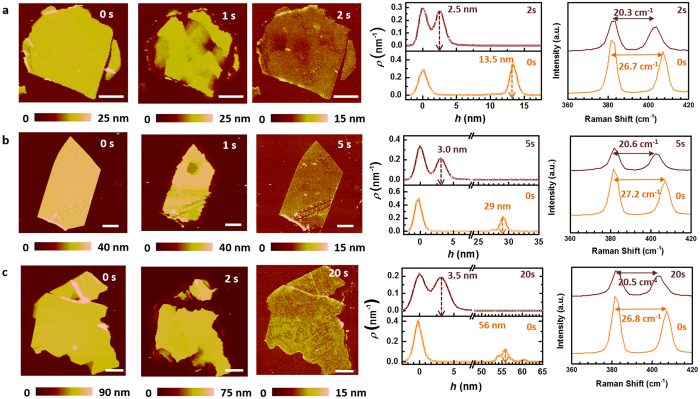
Thickness-dependent time evolution of the ablation of MoS_2_ flakes. AFM images of the flakes of different initial thicknesses: (**a**) 13.5 nm, (**b**) 29 nm, and (**c**) 56 nm. The flakes were treated with the EA technique for different time durations as shown on the right upper corner of the images. The white solid lines on the right lower corners of the images are scale bars of 5 μm, representing their lateral dimensions. The initial (orange solid line) and final (brown solid line) height distributions of the AFM images and the initial (orange solid line) and final (brown solid line) Raman data collected from the flakes are shown in the right most two panels, respectively.

**Figure 4:**
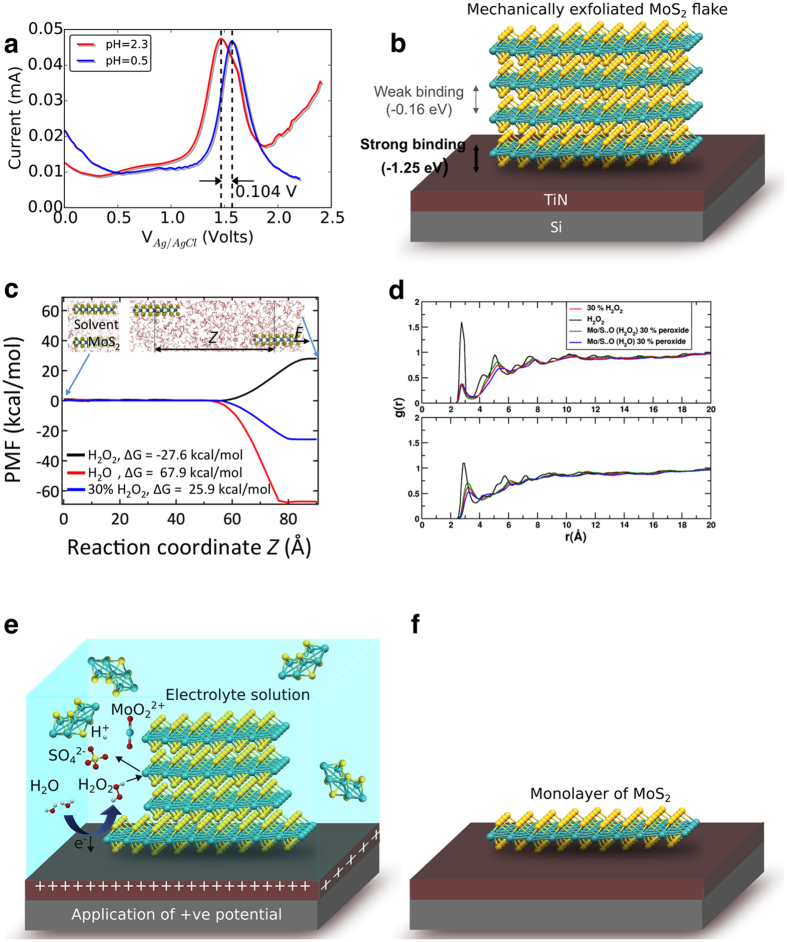
(**a**) Differential pulse voltammetry of the conducting TiN substrate with an electrolyte solution (1 M LiCl) with different pH values. The peak at approx. 1.5 V is attributed to the oxidation of TiN to TiO_2_ and the formation of hydrogen peroxide (H_2_O_2_) from the oxidation of water. (**b**) Our electronic structure calculations suggest that the binding energy *E*_*b*_ = −1.25 eV between the MoS_2_ monolayer and the TiN slab is much stronger than the binding energy between two MoS_2_ monolayers (−0.16 eV). (**c**) Variation in the potential of mean force during the exfoliation of a monolayer from bilayer MoS_2_ in various solvents (H_2_O, H_2_O_2_, 30%H_2_O_2_), as obtained from our ABF-MD simulations. These calculations provide the free energy of exfoliation of MoS_2_, and identify the thermodynamic feasibility of exfoliation in different solvents. (**d**) Pair distribution function between Mo and O of solvents (top panel) and between S and O of solvents (bottom panel). The overall mechanism of monolayer MoS_2_ formation *via* the EA technique is schematically depicted in (**e**,**f**). Mechanically-exfoliated multilayered MoS_2_ flakes undergo etching followed by electrochemical ablation due to the action of H_2_O_2_ produced at the conducting surface of TiN. At the applied electrode potential of 1.5 V, there is oxidation of TiN and H_2_O to form TiO_2_ and H_2_O_2_. The latter is implicated in reactions with the edge/defect sites of MoS_2_ flakes to produce ionic species like SO_4_^2−^, MoO_2_^2+^ and H^+^. These reactions lead to the formation of smaller flakes, which are easier to ablate in an H_2_O_2_ environment. The ablation of smaller flakes continues for all the layers in the solution leaving behind only the layer (f) that is strongly attached to the substrate.

**Figure 5:**
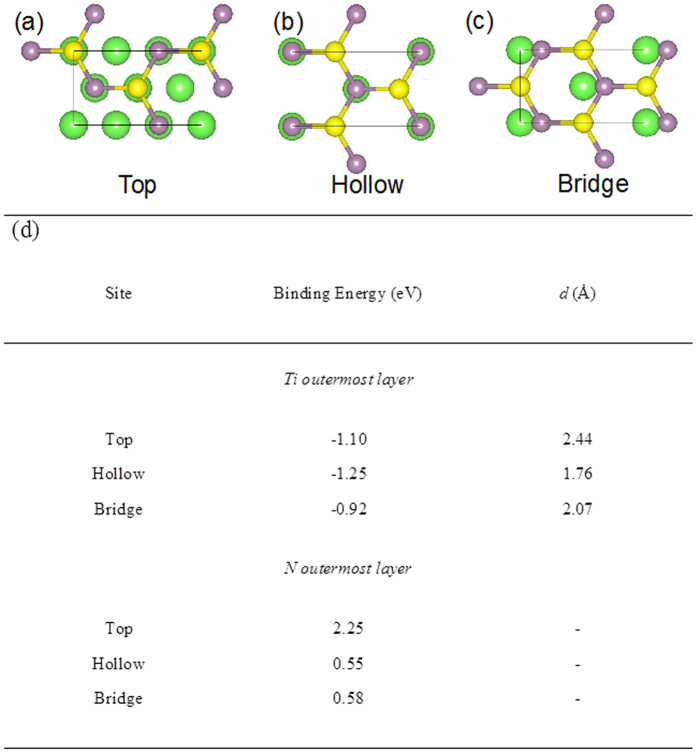
Atomic configurations for MoS_2_ monolayer binding on a TiN (111) slab containing Ti atoms in the outermost layer closest to the S plane of MoS_2_. Three possible sites for attachment of S atoms to the top layer of TiN (111) were investigated, namely (**a**) top, (**b**) hollow, and (**c**) bridge. Only the Ti atoms (green) in the outermost layer of the TiN (111) slab are shown for clarity. The Mo atoms are shown as purple spheres and S atoms are shown in yellow. In addition to these 3 configurations, the TiN (111) slab containing N atoms in the outermost layer were also investigated with the S atoms placed at top, hollow, and bridge sites. (**d**) Table showing the binding energy of MoS_2_ on a TiN (111) slab in various configurations. The closest vertical spacing at equilibrium between the MoS_2_ monolayer and the top layer of the TiN(111) slab *d* is also provided for those configurations that resulted in binding (i.e., negative values of binding energy).

**Figure 6:**
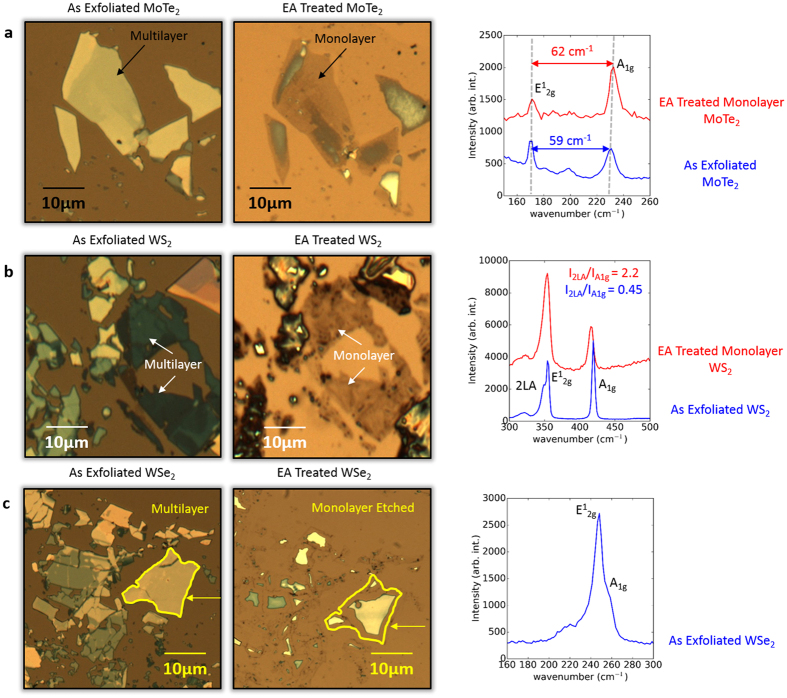
The EA technique applied to different semiconducting TMDs. Optical images and Raman spectra of (**a**) MoTe_2_, (**b**) WS_2_, and (**c**) WSe_2_ flakes before and after the application of the EA technique. Monolayers are successfully obtained for MoTe_2_ (the separation between the A_1g_ and E^1^_2g_ peaks changes from 59 cm^−1^–62 cm^−1^) and WS_2_ (the intensity ratio of the 2LA peak and the A_1g_ peak changes from 0.45–2.2). However, in the case of WSe_2_, the EA technique removes the monolayer as well.
