# Supplementary material for: A Self-Limiting Electro-Ablation Technique for the Top-Down Synthesis of Large-Area Monolayer Flakes of 2D Materials
Source: Sci Rep. 2016 Jun 21;6:28195. doi: 10.1038/srep28195 (PMC4915060; doi:10.1038/srep28195)
Supplement: Supplementary Information [file srep28195-s1.pdf]

Supporting Information

A Self-Limiting Electro-Ablation Technique for the Top-Down Synthesis of Large-Area Monolayer Flakes of 2D Materials

*Saptarshi Das[1, *], Mrinal K. Bera[2, *], Sheng Tong[3], Badri Narayanan[3], Ganesh Kamath[4], Anil Mane[5], Arvydas P. Paulikas[6], Mark R. Antonio[7], Subramanian KRS Sankaranarayanan[3] and Andreas K. Roelofs[3]*

[1]*Department of Engineering Science and Mechanics & Material Research Institute, Pennsylvania State University, State College, 16803, USA*

[2]*DUBBLE-CRG, European Synchrotron Radiation Facility, CS40220, 38043 Grenoble Cedex 9, France*

[3]*Nanoscience and Technology Division, Argonne National Laboratory, Argonne, Illinois, 60439, USA*

[4]*Department of Chemistry, University of Missouri, Columbia, Missouri, 65211, USA*

[5]*Energy Science Division, Argonne National Laboratory, Argonne, Illinois, 60439, USA*

[6]*Material Science Division, Argonne National Laboratory, Argonne, Illinois, 60439, USA*

[7]*Chemical Sciences and Engineering Division, Argonne National Laboratory, Argonne, Illinois, USA*

*Corresponding Author: sud70@psu.edu or das.sapt@gmail.com, mrinal.bera@esrf.fr or nayanbera@gmail.com

Table of Contents

S1. Comparison of electro-ablation (EA) technique with other state-of-the-art techniques
S2. Micromechanical exfoliation – thermally-activated, solvent-mediated, and ultra-sound assisted.
S3. XPS data collected from MoS_2 flakes before and after the application of the EA technique
S4. Ex-situ characterization of a single MoS_2 flake at an intermediate stage of application of the EA technique
S5. Self-limiting aspect of the EA technique
S6: EA mediated by H_2O_2
S7: Passivated TiN Substrate
S8: Electronic structure calculations of binding energetics
S9: Free energy of solvation and potential of mean force calculations

S1: Comparison of the EA technique with other state-of-the-art techniques

Table S1: Comparison of different techniques for large-area synthesis of MoS_2 monolayers						
Approach	Process		Temperature	Time	Yield	Flake Area
Bottom-up	CVD	Sulfurization of Mo[1,2]	750^0 C	~2hrs	-	10^0-10^2 μm^2
		Sulfurization of MoO_3[3-7]	800^0 C	~2hrs	95%	10^0-10^2 μm^2
		Sulfurization of $MoCl_5$[8,9]	850^0 C	~1.5hrs	-	10^0-10^2 μm^2
	MOCVD	Metal-organic CVD[10]	550^0 C	~24hrs	99.5%	10^0-10^2 μm^2
	Thermal	Decomposition of Thiomolybdates[11]	1000^0 C	~2hrs	-	10^{-2}-10^0 μm^2
Top Down	Lithiation and Ultra-sonication[12,13]		100^0 C	~2days	90%	10^{-2}-10^2 μm^2
	Spontaneous Exfoliation (H_2O_2+NMP)[14]		RT	~10hrs	60%	10^{-2}-10^0 μm^2
	Electrochemical Exfoliation (H_2O_2)[15]		RT	~2hrs	7%	10^1-10^3 μm^2
	Electrochemical Exfoliation (Li)[16]		RT	-	92%	10^{-2}-10^0 μm^2
	Shear Exfoliation (Surfactant)[17,18]		RT	~3hrs	17%	10^{-2}-10^0 μm^2
**Top Down**	**EA Technique**		**RT**	**~60secs**	**~100%**	**10^2-10^5 μm^2**

S2: Micromechanical exfoliation – thermally-activated, solvent-mediated, and ultra-sound assisted

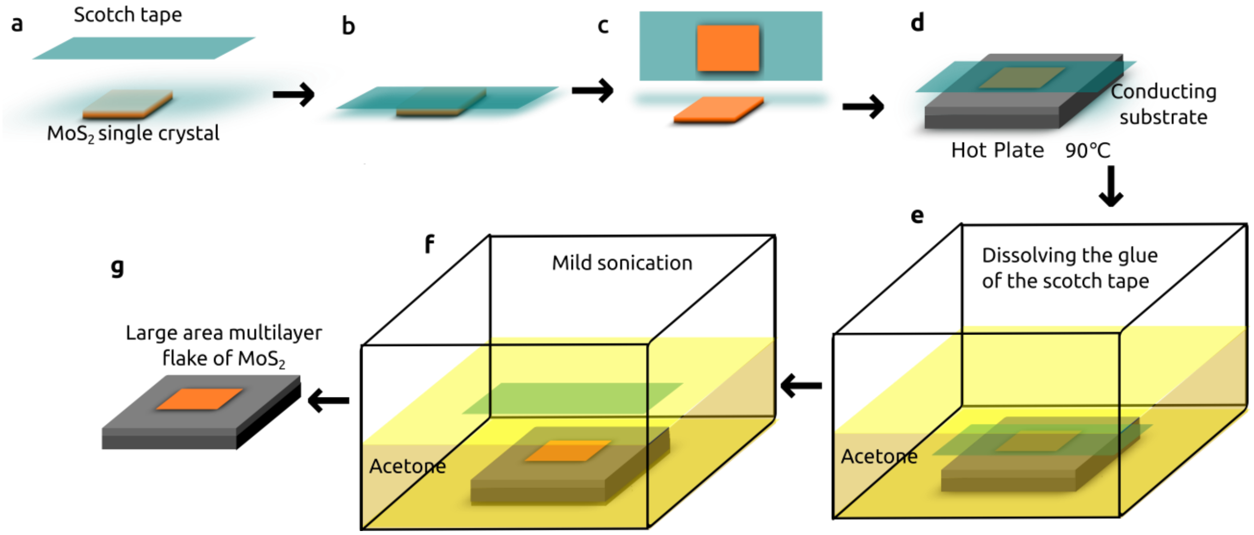

Figure S2. Schematic illustrations of the micromechanical exfoliation method adopted to exfoliate large area flakes on a TiN substrate. (a) First we placed the adhesive tape on a large (1 cm × 1 cm) MoS_2 single crystal. (b) The adhesive tape is pressed down on to the single crystal. (c) The tape is then gently peeled off, resulting in the transfer of large-area multilayer MoS_2 flakes on to the tape. Repeated folding of the tape is avoided to prevent the disintegration of the flakes. (d) The tape with large multilayer flakes is then pressed on to the conductive TiN substrate and placed on a hotplate for 1 minute at 90 ˚C. (e) The taped assembly is then immersed in acetone to dissolve the glue of the tape. (f) Next a mild ultra-sound assisted sonication for less than 5 seconds is applied to peel off the tape leaving behind (g) large-area MoS_2 flakes on the substrate.

S3. XPS data collected from MoS_2 flakes before and after the application of the EA technique

F

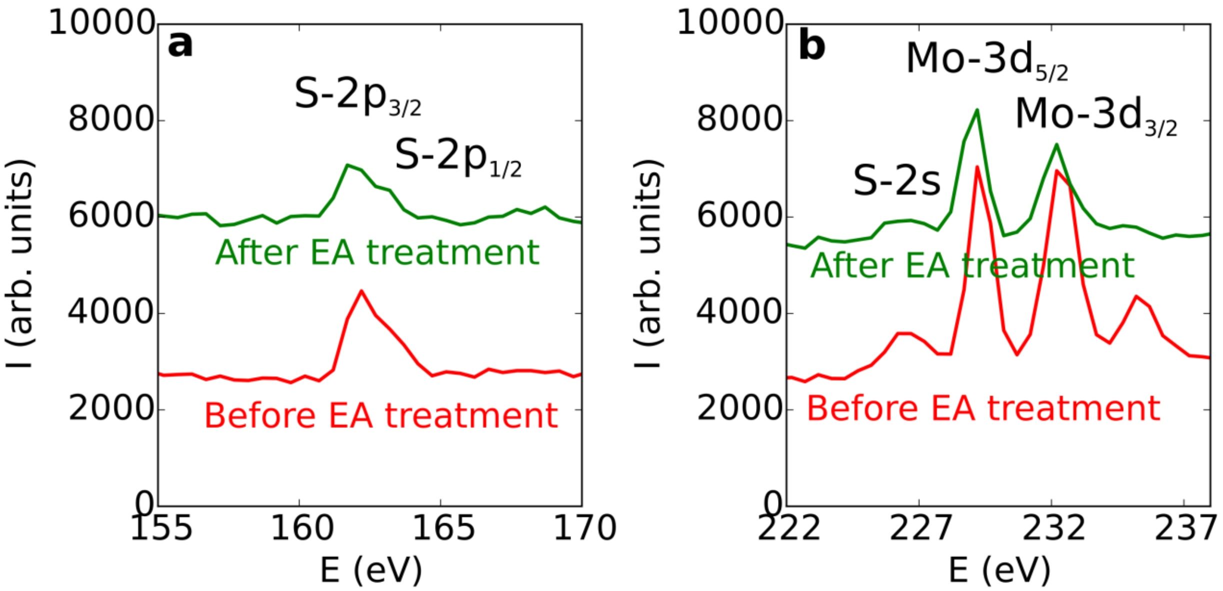

Figure S3. XPS data collected from MoS_2 flakes before and after the application of the EA technique. (a) S-2p (b) Mo-3d peaks from MoS_2 flakes.

S4: Ex-situ characterization of a single MoS_2 flake at an intermediate stage of application of the EA technique

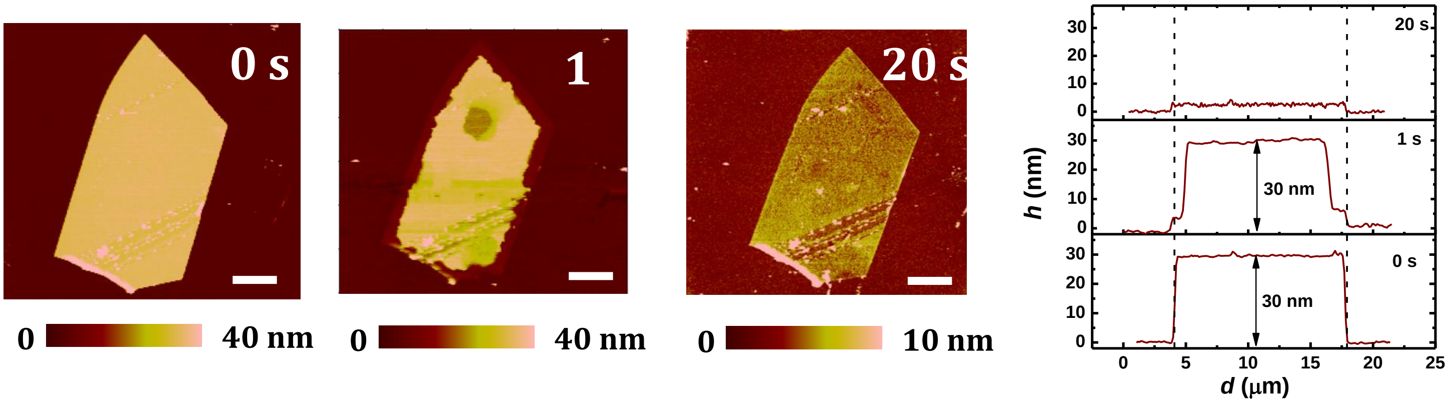

Figure S4. Ex-situ characterization of a single MoS_2 flake at an intermediate stage of etching. (three left panels) AFM images and (rightmost panel) line profiles of a MoS_2 flake after applying the EA technique for 0, 1, and 20 seconds, in which we have both inner unetched (multilayer) and outer etched (monolayer) portions. The white solid lines in the AFM images represent a lateral scale bar of 5 μm.

S5: Self-limiting aspect of the EA technique

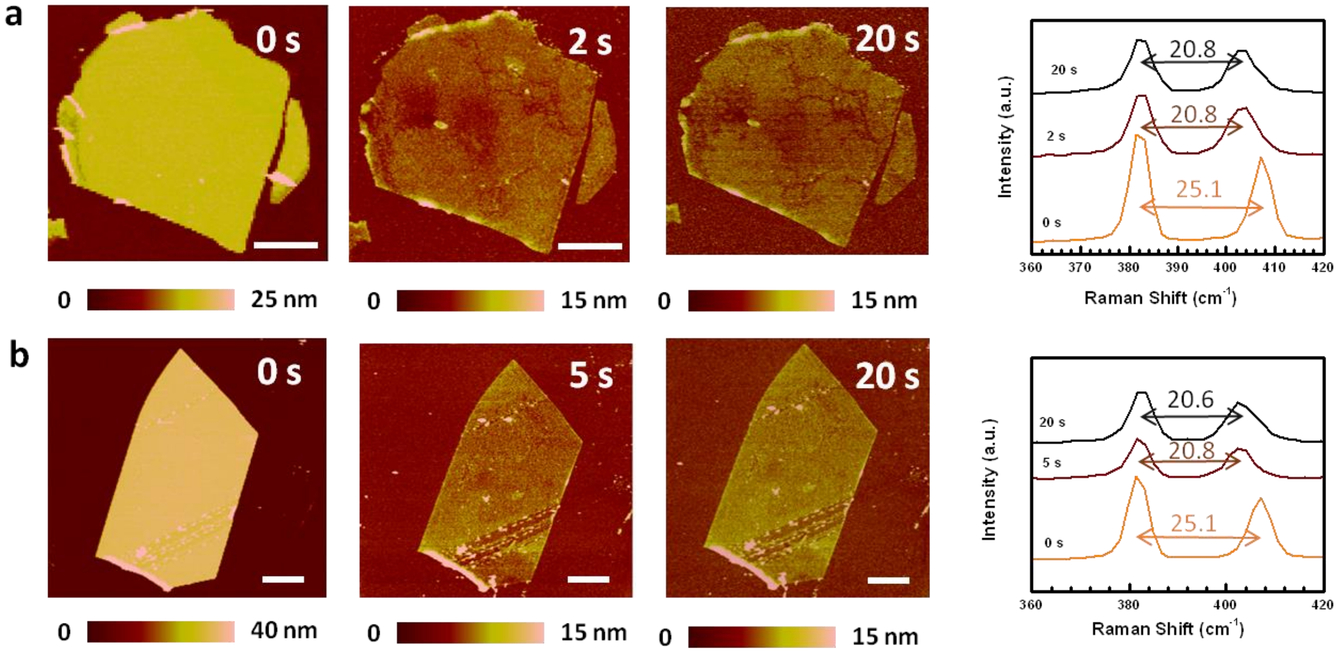

Figure S5. Self-limiting aspect of the EA technique. The time evolution of the AFM images and Raman spectra of the flakes with starting thicknesses of (a) 13.5 nm and (b) 29 nm. For both the flakes, it is evident that once the flakes are ablated to monolayers (Raman shift ~ 20.6 cm^{-1}), the etching process stops or self-limits. The scale bars in all the images are 5 µm.

S6: EA mediated by H_2O_2

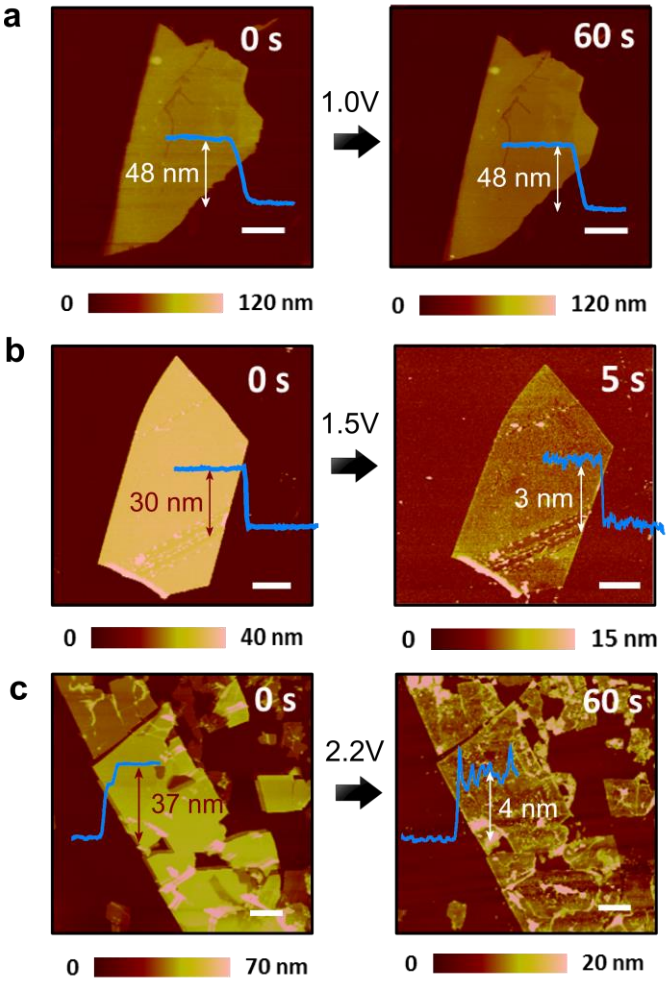

Figure S6. AFM images of MoS_2 flakes before and after the electrochemical treatment performed at different potentials (a) 1.0 V, (b) 1.5 V, and (c) 2.2 V. The line profiles extracted at different edges of the flakes are shown as solid blue lines. Note that the multilayer flakes are converted to monolayers only when the electrode potential is ≥ 1.5 V (oxidation potentials for TiN to TiO_2 and H_2O to H_2O_2 reactions, see text). The scale bars are 5 μm.

S7: Passivated TiN substrate

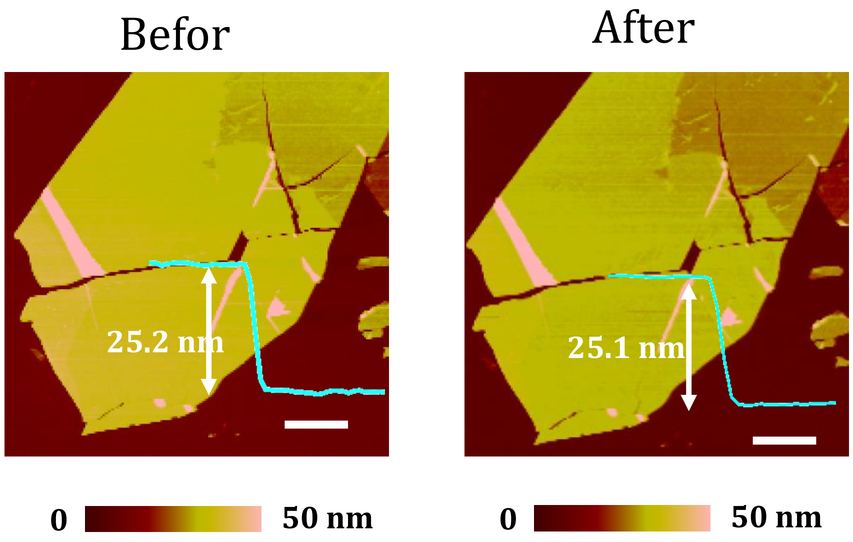

Figure S7. AFM images of MoS_2 flakes exfoliated on a passivated TiN substrate before and after the EA treatment. The height profiles show no changes in thickness of the flakes due to passivation of TiN prior to the adherence of the MoS_2 flakes by the micromechanical exfoliation process (Figure S2). The passivation of TiN was achieved by applying the EA treatment on the bare TiN substrate (without MoS_2 flakes)

S8: Electronic structure calculations of binding energies

We employed density functional theory (DFT) calculations to identify whether it is energetically more favorable for a monolayer of MoS_2 to bind with the TiN substrate rather than another layer of MoS_2. These calculations are performed in the generalized gradient approximation (GGA) using the projector-augmented wave formalism as implemented in the Vienna Ab-initio Simulation Package (VASP).[19,20] The exchange correlation is described by the Perdew-Burke-Ernzerhof (PBE) functional.[21] We verified that the PBE functional reproduces the lattice parameters of monolayer MoS_2, and rocksalt TiN within 1 % of their experimental values; the PBE functional has also been employed successfully in previous investigations on TiN surfaces[22] and MoS_2.[23] The plane wave energy cutoff is set to 500 eV, while the Brillouin zone (BZ) is sampled by a Γ-centered Monkhorst-Pack grid. A k-grid of 8 × 8 × 1 is used for monolayer/bilayer MoS_2, whereas 6 × 10 × 1 is employed for TiN slabs as well as the TiN-MoS_2 configurations. During geometry optimizations, the atomic coordinates are optimized using a conjugate gradient algorithm until the force components on each atom is < 0.01 eV/atom. To describe the van der Waals interactions between two MoS_2 monolayers, we adopted the DFT-D2 method.[24] This technique in conjunction with the PBE functional has been reported to reproduce the experimental interlayer MoS_2 spacing with deviations < 1 %.[25]

To serve as reference for the binding energy calculations, we first optimize the atomic coordinates of (a) a TiN slab (rocksalt crystal structure) oriented with its surface normal pointing along the crystallographic 111 direction, and (b) monolayer MoS_2 in the framework of DFT. For the TiN slab, we employed 9 bilayers of TiN in an orthorhombic (√3×1) supercell, wherein each layer contains 2 Ti or 2 N atoms; the bottom 5 layers are fixed to simulate the bulk. For monolayer MoS_2, we employ an orthorhombic supercell containing 2 Mo and 4 S atoms. Next, we identify the most energetically favorable binding configuration for monolayer MoS_2 on TiN (111) by investigating 6 different configurations: in three of these systems, the Ti layer of the TiN (111) slab is closest to the S of MoS_2, whereas in the remaining three, the N layer of the TiN (111) slab neighbors S of MoS_2. In both these cases (i.e., Ti or N being the surface layer of the TiN slab), the S layer of MoS_2 is placed at three distinct sites in the surface layer of the TiN slab, namely, hollow, top, and bridge (Figure 5). In each of these configurations, a strain of < 4 % is applied to the MoS_2 monolayer to ensure epitaxy with the underlying TiN slab. For each of these 6 configurations, the binding energy (E_b) between the TiN slab and the MoS_2 monolayer is computed as:

$$E_b = E_S - E_{TiN} - E_{MoS_2} \quad (1)$$

Here E_s is the total energy of the relaxed TiN-MoS_2 system in one of the 6 different configurations, while E_{TiN} and E_{MoS_2} refer to the total energies of the optimized TiN slab and the MoS_2 monolayer. Similarly, the binding energy between two MoS_2 monolayers is given by:

$$E_b = E_S - 2E_{MoS_2} \quad (2)$$

Here E_s is the total energy of the MoS_2 bilayer (following the stacking sequence in bulk MoS_2).

Our computed binding energies for the various configurations are shown in Table 1. As indicated by Table 1, we find that the binding energy E_b is highest (most negative) when the MoS_2 monolayer binds onto a TiN slab containing Ti atoms in its outermost layer, such that the closest S atoms in MoS_2 lie in the hollow sites. In this configuration, there is a strong covalent interaction between MoS_2 and TiN as indicated by the value of E_b = -1.25 eV. In comparison, our DFT calculations show that the binding energy between two MoS_2 monolayers is much lower (-0.16 eV) owing to weak van der Waals interactions. Evidently, it is energetically preferable for a monolayer of MoS_2 to bind to the underlying TiN substrate rather than to another MoS_2 layer.

S9: Free energy of solvation and potential of mean force calculations

To provide a thermodynamic basis towards the observed phenomenon, we employed the Adaptive Bias Force method[25] implemented in NAMD version 2.9[26] to determine the free energies of exfoliation and dispersion of MoS_2 in water and hydrogen peroxide. Details of the ABF algorithm and implementation can be found in Refs.[27,28]. As an initial model construct, we started with a bilayer MoS_2 sheet [inset Figure S9]. As a part of the ABF algorithm, an external biasing force, estimated locally from the sampled conformations of the system and updated continuously, is applied at each step to facilitate the system in overcoming significant energy barriers, if present, along the reaction coordinate. This force is applied to one of the MoS_2 sheets in the *z*-direction, such that the sheets are tangentially separated. An orthorhombic computational supercell was employed with dimensions of 80 Å × 80 Å × 200 Å; the heterogeneous condensed phase occupies an 80 Å × 80 Å × 80 Å region. The number of solvent molecules in each box was selected to reproduce the density of water or hydrogen peroxide by isothermal–isobaric (NPT) simulations at 1 atm and 300 K. A sufficiently large cut-off distance of 14 Å is employed. Initial configurations for each system were generated using Packmol; subsequently the atomic coordinates were optimized for 5000 steps via a steepest descent algorithm followed by MD simulations. During these MD runs, systems were equilibrated over a time period of 10 ns in the NVT ensemble, with another production of 20 ns, followed by the ABF calculation in the NVT canonical ensemble for 30 ns for each window (6 windows of 15 Å). The MoS_2 sheets were based on the potential of Farmer and co-workers[29] while SPC/E force field[30] was used for water and AMBER modified potential for hydrogen peroxide, respectively.

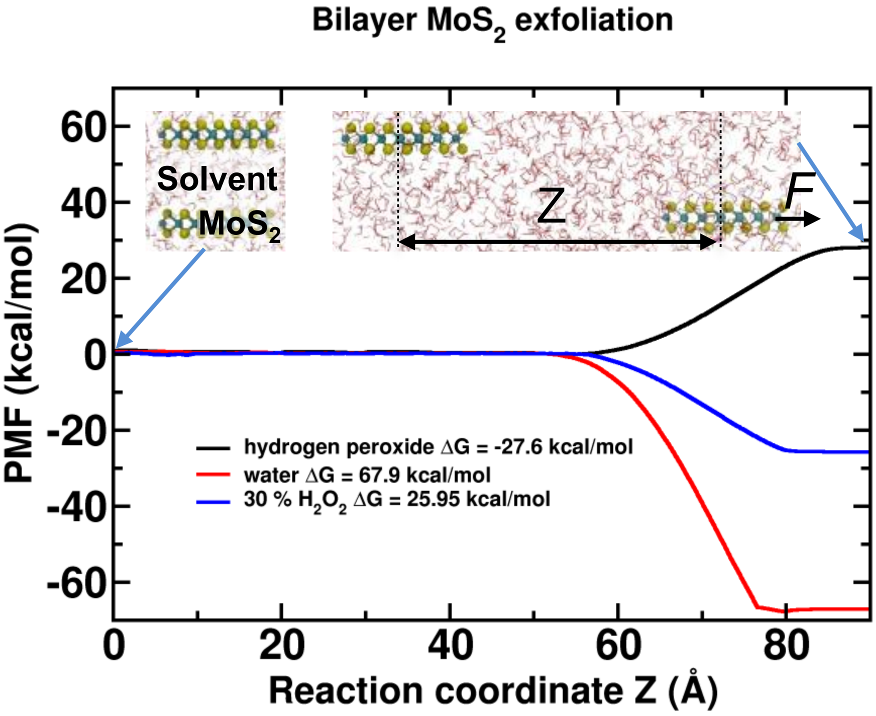

Figure S9. ABF-MD calculations showing the variation in potential of mean force (kcal/mol) in various solvents. The initial (left) and the final (right) configurations for a typical run are shown as insets.

References

1 Song, I. *et al.* Patternable Large-Scale Molybdenium Disulfide Atomic Layers Grown by Gold-Assisted Chemical Vapor Deposition. *Angew. Chem. Int. Ed.* **53**, 1266-1269, doi:10.1002/anie.201309474 (2014).

2 Zhan, Y., Liu, Z., Najmaei, S., Ajayan, P. M. & Lou, J. Large-Area Vapor-Phase Growth and Characterization of MoS_2 Atomic Layers on a SiO_2 Substrate. *Small* **8**, 966-971 (2012).

3 van der Zande, A. M. *et al.* Grains and grain boundaries in highly crystalline monolayer molybdenum disulphide. *Nat. Mater.* **12**, 554-561, doi:10.1038/nmat3633 (2013).

4 Dumcenco, D. *et al.* Large-Area Epitaxial Mono layer MoS_2. *ACS Nano* **9**, 4611-4620, doi:10.1021/acsnano.5b01281 (2015).

5 Liu, H. *et al.* Statistical study of deep submicron dual-gated field-effect transistors on monolayer chemical vapor deposition molybdenum disulfide films. *Nano Lett.* **13**, 2640-2646, doi:10.1021/nl400778q (2013).

6 Lee, Y.-H. *et al.* Synthesis of Large-Area MoS_2 Atomic Layers with Chemical Vapor Deposition. *Adv. Mater.* **24**, 2320-2325 (2012).

7 Najmaei, S. *et al.* Vapour Phase Growth and Grain Boundary Structure of Molybdenum Disulphide Atomic Layers. *Nat. Mater.* **12**, 754-759, doi:10.1038/nmat3673 (2013).

8 Yu, Y. *et al.* Controlled Scalable Synthesis of Uniform, High-Quality Monolayer and Few-layer MoS_2 Films. *Sci. Rep.* **3** (2013).

9 McCreary, K. M. *et al.* Large-Area Synthesis of Continuous and Uniform MoS_2 Monolayer Films on Graphene. *Adv. Funct. Mater.* **24**, 6449-6454, doi:10.1002/adfm.201401511 (2014).

10 Kang, K. *et al.* High-Mobility Three-Atom-Thick Semiconducting Films with Wafer-Scale Homogeneity. *Nature* **520**, 656-660, doi:10.1038/nature14417 (2015).

11 Liu, K.-K. *et al.* Growth of Large-Area and Highly Crystalline MoS_2 Thin Layers on Insulating Substrates. *Nano Lett.* **12**, 1538-1544, doi:10.1021/nl2043612 (2012).

12 Matte, H. S. S. R. *et al.* MoS_2 and WS_2 Analogues of Graphene. *Angew. Chem. Int. Ed.* **49**, 4059-4062, doi:10.1002/anie.201000009 (2010).

13 Zheng, J. *et al.* High Yield Exfoliation of Two-Dimensional Chalcogenides Using Sodium Naphthalenide. *Nat. Commun.* **5**, 2995 (2014).

14 Dong, L. *et al.* Spontaneous Exfoliation and Tailoring of MoS_2 in Mixed Solvents. *Chem. Commun.* **50**, 15936-15939 (2014).

15 Liu, N. *et al.* Large-Area Atomically Thin MoS_2 Nanosheets Prepared Using Electrochemical Exfoliation. *ACS Nano* **8**, 6902-6910 (2014).

16 Zeng, Z. *et al.* Single-Layer Semiconducting Nanosheets: High-Yield Preparation and Device Fabrication. *Angew. Chem. Int. Ed.* **50**, 11093-11097 (2011).

17 Varrla, E. *et al.* Large-Scale Production of Size-Controlled MoS_2 Nanosheets by Shear Exfoliation. *Chem. Mater.* **27**, 1129-1139, doi:10.1021/cm5044864 (2015).

18 Coleman, J. N. *et al.* Two-Dimensional Nanosheets Produced by Liquid Exfoliation of Layered Materials. *Science* **331**, 568-571, doi:10.1126/science.1194975 (2011).

19 Kresse, G. & Furthmuller, J. Efficient iterative schemes for ab initio total-energy calculations using a plane-wave basis set. *Phys. Rev. B* **54**, 11169-11186, doi:DOI 10.1103/PhysRevB.54.11169 (1996).

20 Kresse, G. & Furthmuller, J. Efficiency of ab-initio total energy calculations for metals and semiconductors using a plane-wave basis set. *Comp. Mater. Sci.* **6**, 15-50, doi:Doi 10.1016/0927-0256(96)00008-0 (1996).

21 Perdew, J. P., Burke, K. & Ernzerhof, M. Generalized gradient approximation made simple. *Phys. Rev. Lett.* **77**, 3865-3868, doi:DOI 10.1103/PhysRevLett.77.3865 (1996).

22 Marlo, M. & Milman, V. Density-functional study of bulk and surface properties of titanium nitride using different exchange-correlation functionals. *Phys. Rev. B* **62**, 2899-2907, doi:DOI 10.1103/PhysRevB.62.2899 (2000).

23 Peelaers, H. & Van de Walle, C. G. First-principles study of van der Waals interactions in MoS_2 and MoO_3. *J. Phys.-Condens. Mat.* **26**, doi:Artn 305502
10.1088/0953-8984/26/30/305502 (2014).

24 Grimme, S. Semiempirical GGA-type density functional constructed with a long-range dispersion correction. *J. Comput. Chem.* **27**, 1787-1799, doi:10.1002/jcc.20495 (2006).

25 Henin, J., Fiorin, G., Chipot, C. & Klein, M. L. Exploring Multidimensional Free Energy Landscapes Using Time-Dependent Biases on Collective Variables. *J. Chem. Theory Comput.* **6**, 35-47, doi:10.1021/ct9004432 (2010).

26 Phillips, J. C. *et al.* Scalable molecular dynamics with NAMD. *J. Comput. Chem.* **26**, 1781-1802, doi:10.1002/jcc.20289 (2005).

27 Darve, E., Rodríguez-Gómez, D. & Pohorille, A. Adaptive Biasing Force Method for Scalar and Vector Free Energy Calculations. *J. Chem. Phys.* **128**, 144120 (2008).

28 Darve, E. & Pohorille, A. Calculating free energies using average force. *J. Chem. Phys.* **115**, 9169-9183, doi:Doi 10.1063/1.1410978 (2001).

29 Varshney, V. *et al.* MD simulations of molybdenum disulphide (MoS_2): Force-field parameterization and thermal transport behavior. *Comp. Mater. Sci.* **48**, 101-108, doi:10.1016/j.commatsci.2009.12.009 (2010).

30 Berendsen, H. J. C., Grigera, J. R. & Straatsma, T. P. The Missing Term in Effective Pair Potentials. *J. Phys. Chem.* **91**, 6269-6271, doi:DOI 10.1021/j100308a038 (1987).
